# Deletion of *Transmembrane protein 184b* leads to retina degeneration in mice

**DOI:** 10.1111/cpr.13751

**Published:** 2024-10-07

**Authors:** Guo Liu, Tiannan Liu, Junkai Tan, Xiaoyan Jiang, Yudi Fan, Kuanxiang Sun, Wenjing Liu, Xuyang Liu, Yeming Yang, Xianjun Zhu

**Affiliations:** ^1^ The Sichuan Provincial Key Laboratory for Human Disease Gene Study and Center for Medical Genetics, Sichuan Provincial People's Hospital University of Electronic Science and Technology of China Chengdu Sichuan China; ^2^ Henan Branch of National Clinical Research Center for Ocular Diseases, Henan Eye Hospital People's Hospital of Zhengzhou University, Henan Provincial People's Hospital Zhengzhou Henan China; ^3^ Xiamen Eye Center, Xiamen Research Center for Eye Diseases and Key Laboratory of Ophthalmology Xiamen University Xiamen Fujian China; ^4^ Qinghai Key Laboratory of Qinghai Tibet Plateau Biological Resources, Chinese Academy of Sciences and Qinghai Provincial Key Laboratory of Tibetan Medicine Research Northwest Institute of Plateau Biology Xining Qinghai China; ^5^ Research Unit for Blindness Prevention of Chinese Academy of Medical Sciences (2019RU026) Sichuan Academy of Medical Sciences and Sichuan Provincial People's Hospital Chengdu Sichuan China

## Abstract

*Transmembrane protein 184b* (*Tmem184b*) has been implicated in axon degeneration and neuromuscular junction dysfunction. Notably, Tmem184b exhibits high expression levels in the retina; however, its specific function within this tissue remains poorly understood. To elucidate the role of *Tmem184b* in the mammalian visual system, we developed a *Tmem184b* knockout (KO) model for further investigation. Loss of *Tmem184b* led to significant decreases in both a and b wave amplitudes of scotopic electroretinogram (ERG) and reduced b wave amplitudes of photopic ERG, respectively, reflecting damage to both the photoreceptors and secondary neuronal cells of the retina. Histologic analyses showed a progressive retinal thinning accompanied by the significantly loss of retinal cells including cone, rod, bipolar, horizontal and retinal ganglion cells. The expression levels of photo‐transduction‐related proteins were down‐regulated in KO retina. TUNEL (terminal deoxynucleotidyl transferase‐mediated biotinylated Uridine‐5'‐triphosphate [UTP] nick end labelling) and glial fibrillary acidic protein (GFAP)‐labelling results suggested the increased cell death and inflammation in the KO mice. RNA‐sequencing analysis and GO enrichment analysis revealed that *Tmem184b* deletion resulted in down‐regulated genes involved in various biological processes such as visual perception, response to hypoxia, regulation of transmembrane transporter activity. Taken together, our study revealed essential roles of *Tmem184b* in the mammalian retina and confirmed the underlying mechanisms including cell death, inflammation and hypoxia pathway in the absence of *Tmem184b*, providing a potential target for therapeutic and diagnostic development.

## Graphical abstract

Our study characterized the retinal phenotypes of *Tmem184b* KO mice and confirmed the underlying mechanisms including cell death, inflammation and response to hypoxia pathway for retinal degeneration (RD) in the absence of TMEM184B, providing a rationale for therapeutic and diagnostic applications of RD.

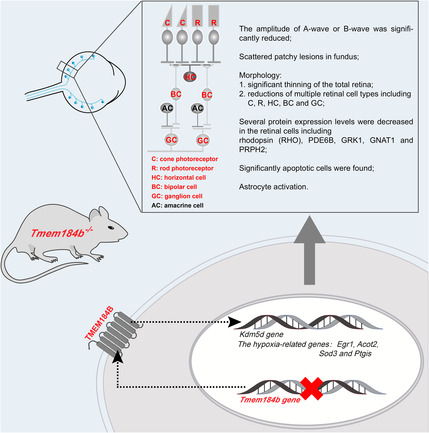

## 1 INTRODUCTION

Retinal degenerative diseases (RD), including age‐related macular degeneration (AMD), retinitis pigmentosa (RP) and glaucoma etc., are most common forms of inheritable RD globally.^1^ Irreversible neural damage in RD leads to irreversible visual impairment or blindness, due to lack of regeneration capacity in adult mammalian central nervous system (CNS).^2^ AMD, RP and glaucoma, the leading cause of irreversible blindness share common pathogenic pathways such as inflammation, oxidative stress and impaired autophagy,^3^, ^4^, ^5^ in which, oxidative stress is one of the most important factors due to high oxygen consumption of retinal cells.^6^ Various innovative therapeutics such as gene and cell therapies are developing to cure them based on these common pathogenic pathways.^7^ As a basic tool, appropriate animal models with clear genetic backgrounds and pathogenic mechanisms are seriously needed in these developments. Genetically engineered mouse models mimicking retinal degenerating diseases have been widely used in ophthalmic researching frontiers worldwide.^8^, ^9^

*Transmembrane protein 184B* gene (*Tmem184b*) encodes a seven‐transmembrane protein which was still poorly characterized. Bioinformatics analysis revealed *Tmem184b* was associated with mammographic density^10^ and breast cancer risk.^11^ Several reports of *Tmem184b* were published mainly focusing on tumour genesis,^12^, ^13^, ^14^ immune related glomerulonephritis and interleukin‐31‐induced itch,^15^, ^16^ coronary artery disease^17^ and axon degenerative central nervous system (CNS) dysfunction.^18^, ^19^, ^20^ Additionally, it has been identified as a putative modulator of ibuprofen and taurine transport.^21^ Interestingly, TMEM184B has been reported to be involved in axon degeneration and neuromuscular junction maintenance,^18^ restraining ectopic firing by ensuring proper locomotion at the neuromuscular junction in *Drosophila* ^19^ and promoting expression of synaptic gene networks in the mouse hippocampus.^20^ As a part of CNS, the mammalian retina shares same principle and pathogenic pathways with nervous system,^22^ and *Tmem184b* gene may play similar roles in retinal diseases. Moreover, *Tmem184b* gene is expressed in the retina, especially in oligodendrocytes, astrocytes, Müller glia cells and excitatory neurons (https://www.proteinatlas.org/ENSG00000198792-TMEM184B/single+cell+type/eye). However, the role of *Tmem184b* in the visual system remains elusive.

To assess the role of *Tmem184b* in vivo, we generated a *Tmem184b* knockout (KO) mouse model by clustered regularly interspaced short palindromic repeats/CRISPR associated 9 (CRISPR/Cas9). Loss of *Tmem184b* led to diminished amplitudes in both a and b waves of scotopic electroretinogram (ERG) analysis, indicating impaired photoreceptor function. Progressive retinal degeneration was observed in *Tmem184b* KO mice. Our study demonstrated an essential role of *Tmem184b* in maintenance of retinal function, providing a potential target for therapeutic and diagnostic development of RD.

## 2 RESULTS

### 2.1 Generation of *Tmem184b* KO model

**FIGURE 1:**
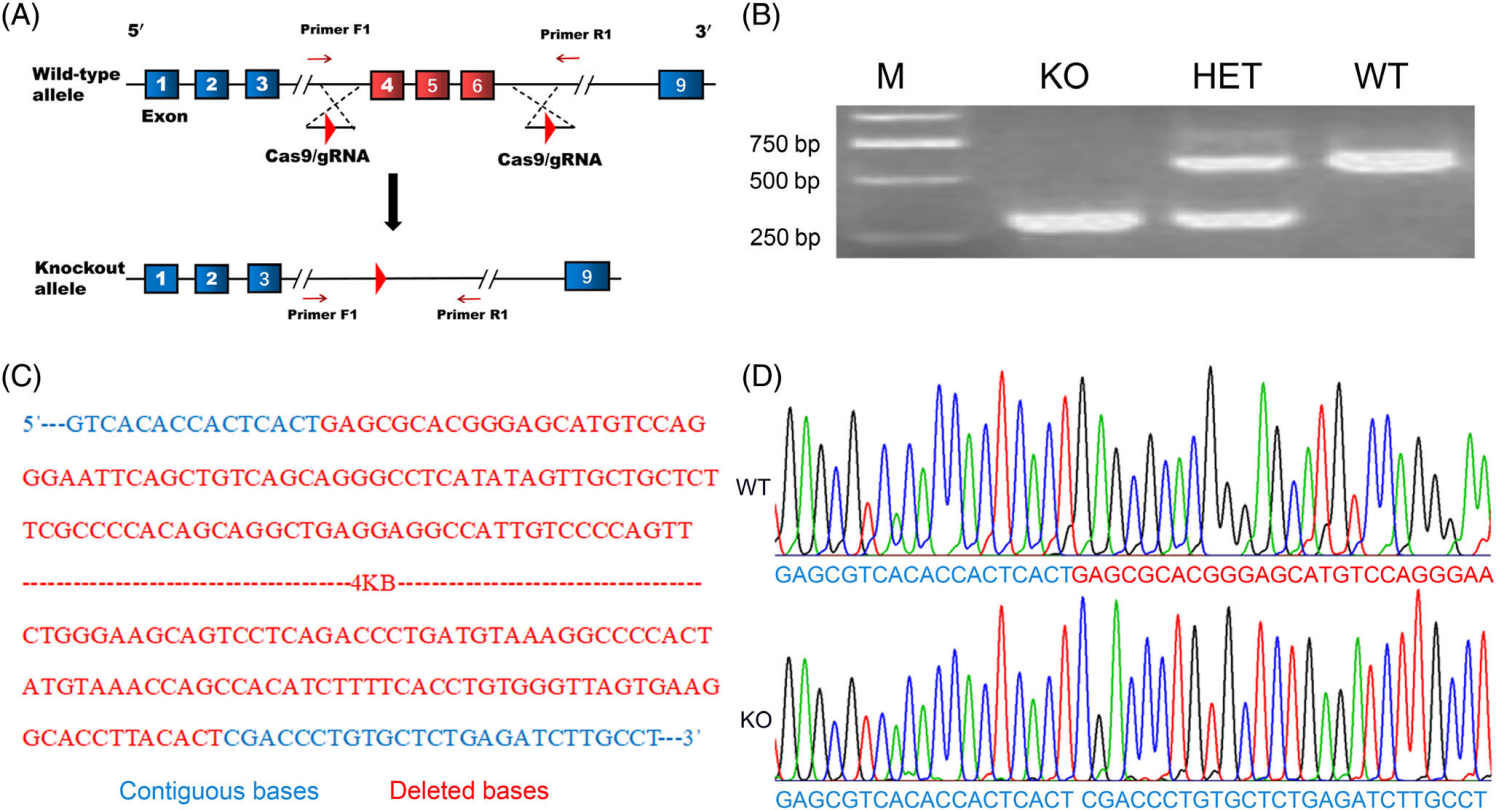
Knockout (KO) strategy and verification by electrophoresis and Sanger sequencing analysis of *Tmem184b* KO model. (A) Knock out strategy, squares in red and blue indicate exons to be knocked out or kept, respectively; dashed line showed the guide RNA designed based on CRISPR/Cas9 technology; red arrows stand for the positions of identifying primers. (B) Identifying agarose gel electrophoresis photo of mouse tail tip genome DNA amplified PCR products. (C) Schematic diagram of the knocked out region. (D) Identifying sequencing peak plot of mouse tail tip genome DNA amplified PCR products; knocked out base pairs were showed in red. M, marker; HET, heterozygous; WT, wild type.

*Tmem184b* KO model was generated using CRISPR/cas9 technology. A genomic deletion of 4002 base pairs (bp) was generated in the KO allele (Figure 1A). Mice carrying KO alleles were backcrossed to C57BL/6J mice for five generations and then inter‐crossed to generate homozygous *Tmem184b* KO mice. Genome DNA polymerase chain reaction (PCR) analysis was used to genotype *Tmem184b* KO with agarose gel electrophoresis (Figure 1B) and Sanger sequencing (Figure 1C,D). As showed in Figure 1B, different PCR products were amplified from homozygotes (HOM, one band of 270 bp), heterozygote (HET, two bands of 270 and 720 bp) and wild type (WT, one band of 720 bp), respectively. Additionally, Sanger sequencing of PCR products confirmed a deletion of 4002 bp in the genome (Figure 1C,D). These data confirmed that *Tmem184b* was successfully deleted in *Tmem184b* KO model.

### 2.2 Deletion of *Tmem184b* led to impaired visual function

**FIGURE 2:**
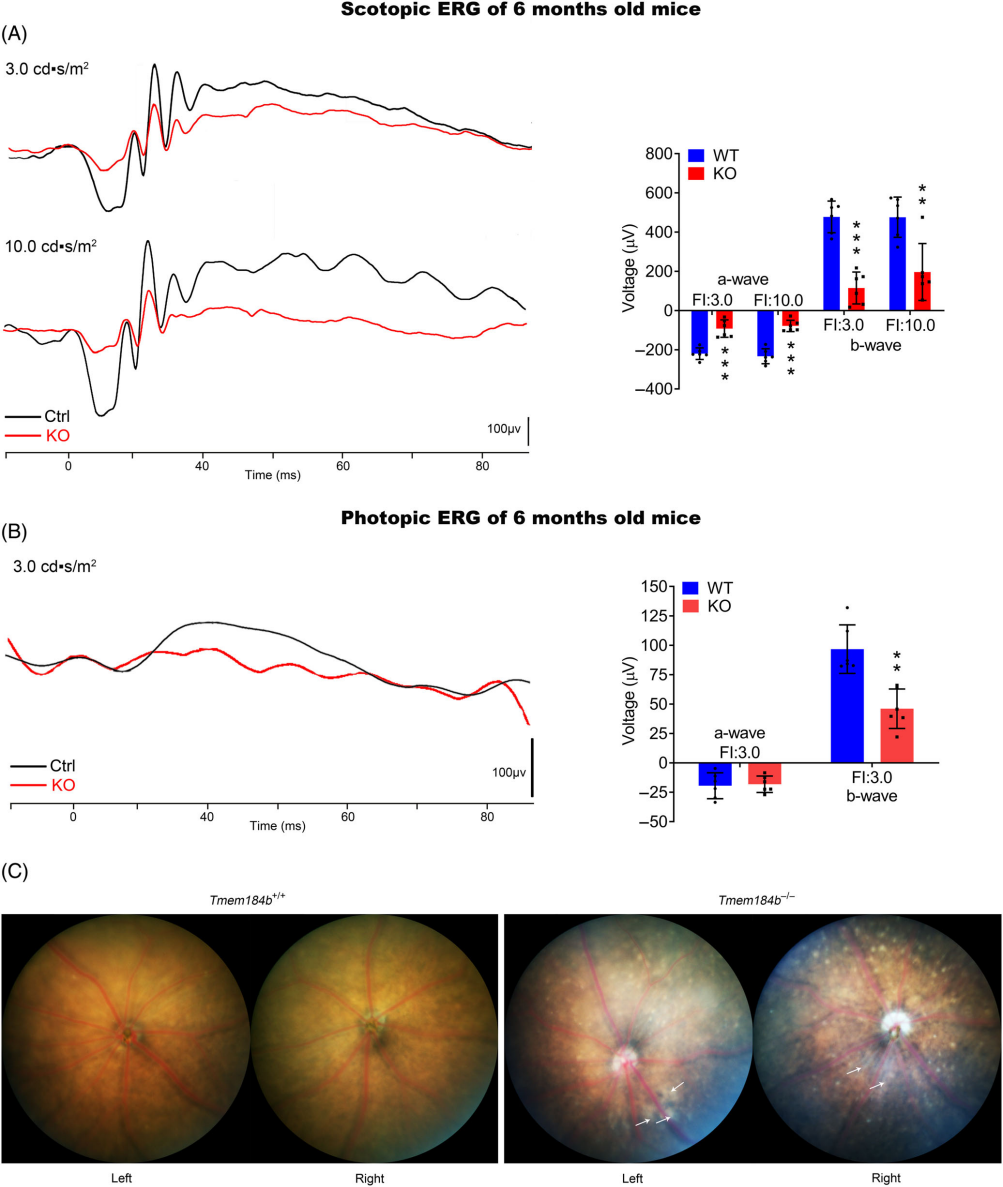
Electroretinogram (ERG) and fundus imaging revealed visual impairments in *Tmem184b* knockout (KO) mice. (A) Dark‐adapted ERG waveform diagrams (left) and corresponding statistical charts of mean amplitudes (right) were shown. (B) Light‐adapted ERG waveform diagrams (left) and corresponding statistical charts of mean amplitudes (right) were shown. (C) Fundus imaging photos of wild‐type (WT) (left) and KO mice. White arrows indicate grey scattered patchy lesions.

To evaluate the physiological effect of *Tmem184b* deficiency, 6‐month‐old *Tmem184b* KO mice were subjected to ERG test. As showed in Figure 2, KO mice exhibited lower mean amplitudes of a‐ and b‐ waves than their WT littermates in dark adapted scotopic ERG tests (Figure 2A). Meanwhile, in light adapted photopic condition, only mean amplitudes of b‐wave were reduced to approximately 50% of their littermate controls (Figure 2B). Moreover, pigment deposits and grey scattered patchy lesions were evident in fundus imaging of *Tmem184b* KO mice, while WT littermates exhibited clear and normal fundus appearance (Figure 2C). Optical coherence tomography (OCT) was used to assess detailed retina structure of *Tmem184b* KO model. More high reflection points were found in ganglion cell layer (GCL) of KO retinas than in WT controls (Figure S2). No other significant change was observed (Figure S1).

Rotarod test was used to investigate if there was any motor deficits or other secondary deficits caused by *Tmem184b* ablation. Six months old KO mice did not show significant motor deficits (Figure S2C). No visible pathological changes were observed in the cerebellum sections (Figure S2B).

### 2.3 *Tmem184b* KO mice exhibited progressive retinal degeneration

**FIGURE 3:**
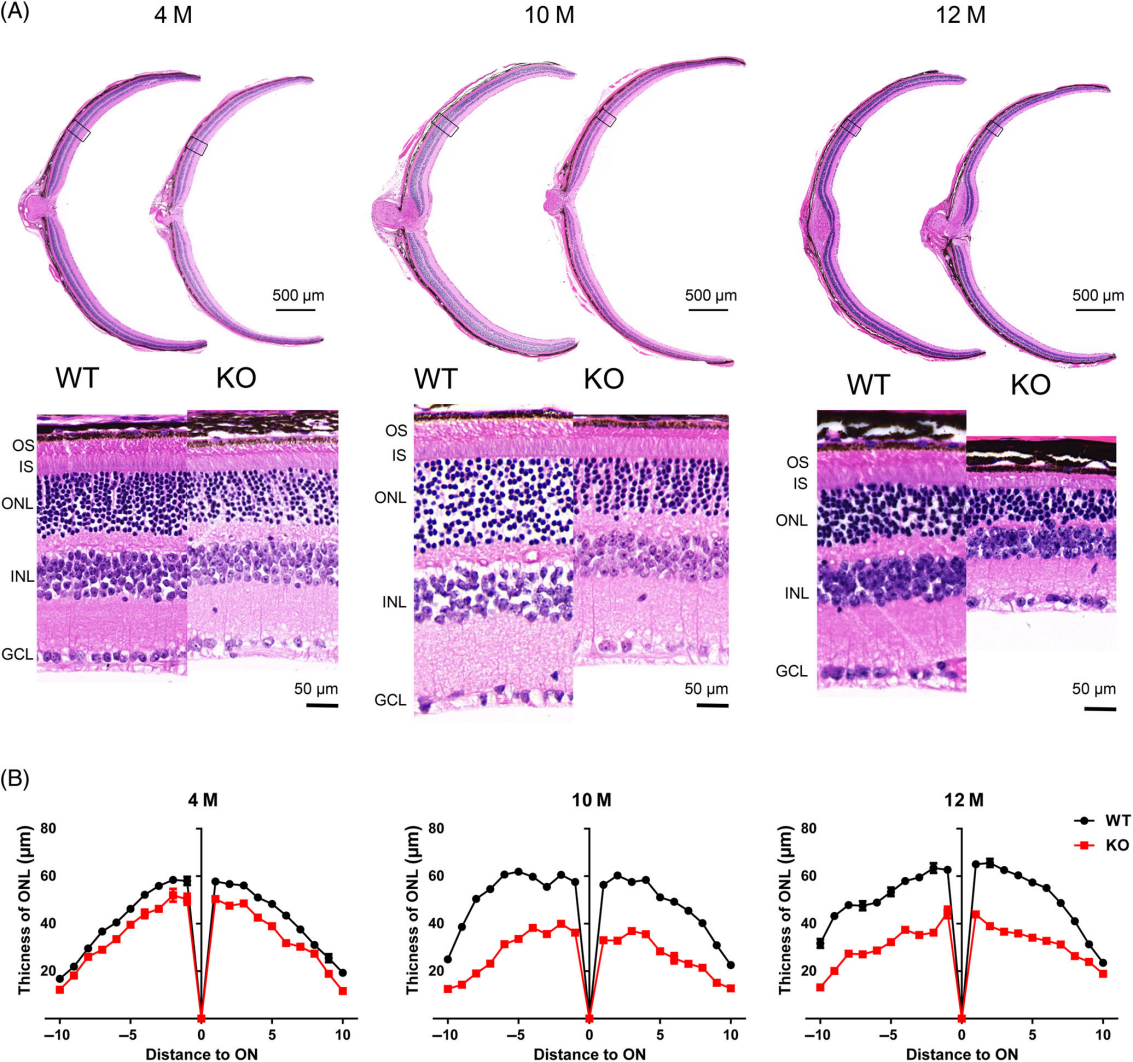
Progressive retinal degeneration in *Tmem184b* knockout (KO) mice. (A) Haematoxylin and eosin (H&E) staining on paraffin sections of the KO and corresponding wild‐type (WT) retinas at the ages of 4, 10 and 12 months. Squares on full sized retinas (up) showed the intercepted and zoomed in scopes of the retinas (down). Scale bars: 500 μm (up) for original view and 50 μm (down) for zoomed in scope. (B) Thickness analysis of the ONL (*n* = 3). M, months, OS, outer segment, IS, inner segment, ONL, outer nuclear layer, INL, inner nuclear layer; GCL, ganglion cells layer, ON, optic nerve head.

After euthanasia, eyeballs were collected for paraffin sections at different ages to evaluate retinal pathological change. Progressive degeneration of retinal neurons in *Tmem184b* was evident in KO retinas. As showed in Figure 3A, the thickness of KO retinas was reduce to about half of WT mice at 10 months of age (Figure 3A). Statistical analysis showed that KO outer segment layer (ONL) thickness was progressively reduced from about 50 μm at 4 months of age to approximate 30 μm at 12 month of age (Figure 3B). These data indicated progressive retinal degeneration upon *Tmem184b* deletion. Additionally, to assess if there was any structural change in cerebellum, cerebellar paraffin sections haematoxylin and eosin (H&E) staining was performed (Figure S2B). No significant structural change in cerebellum was found.

### 2.4 Severe pathological changes in photoreceptor cells were detected in *Tmem184b* KO mice

**FIGURE 4:**
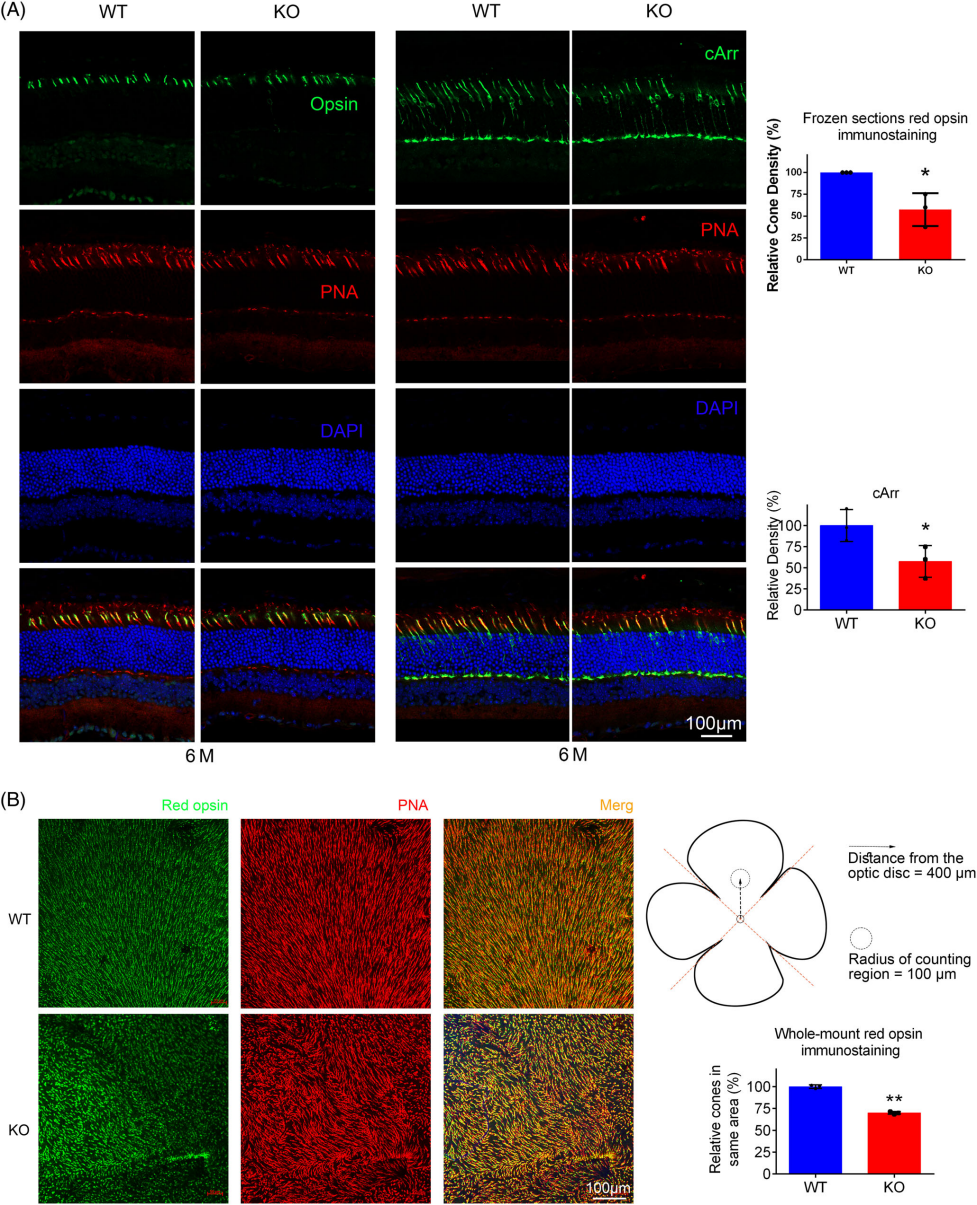
Immunofluorescence (IF) staining (cone) revealed *Tmem184b* knockout (KO) mice suffered from severe photoreceptor degradation. (A) IF staining on frozen sections showed cell morphological changes of photoreceptor. Anti‐L/M Opsin (Opsin) and cone Arrestin (cArr) were used to label cones, Alexa Fluor™ 594 conjugated PNA (PNA) was used to stain cell bodies of cone photoreceptors. Cone‐related statistical charts were arranged in the right panel. (B) IF staining on retinal whole mounts and related cone density count revealed cone degradation. The schematic diagram on up right side showed the same counting regions on each group. The statistical chart under the schematic diagram exhibited the relative cone densities of WT and KO, respectively. Data were presented as mean ± SEM. **p < 0.01, *p < 0.05.

Immunofluorescence (IF) staining was performed on retinal frozen sections and retina flat mounts obtained from 6‐month‐old tested mice. Anti‐L/M opsin and anti‐coe arrestin (cArr) antibodies were used to label cones, while anti‐Rhodopsin (1D4) and anti‐peripherin 2 (PRPH2) antibodies were used to mark rods. Results showed that cone cells of KO, labelled by cArr and opsin, became sparser comparing to those of WT (Figure 4A, left). Opsin marked cones on retinal whole mount revealed reduced cones density (Figure 4B). Statistical analysis of cone density and the schematic diagram of counting area on retinal whole mounts were showed in the right panel.

**FIGURE 5:**
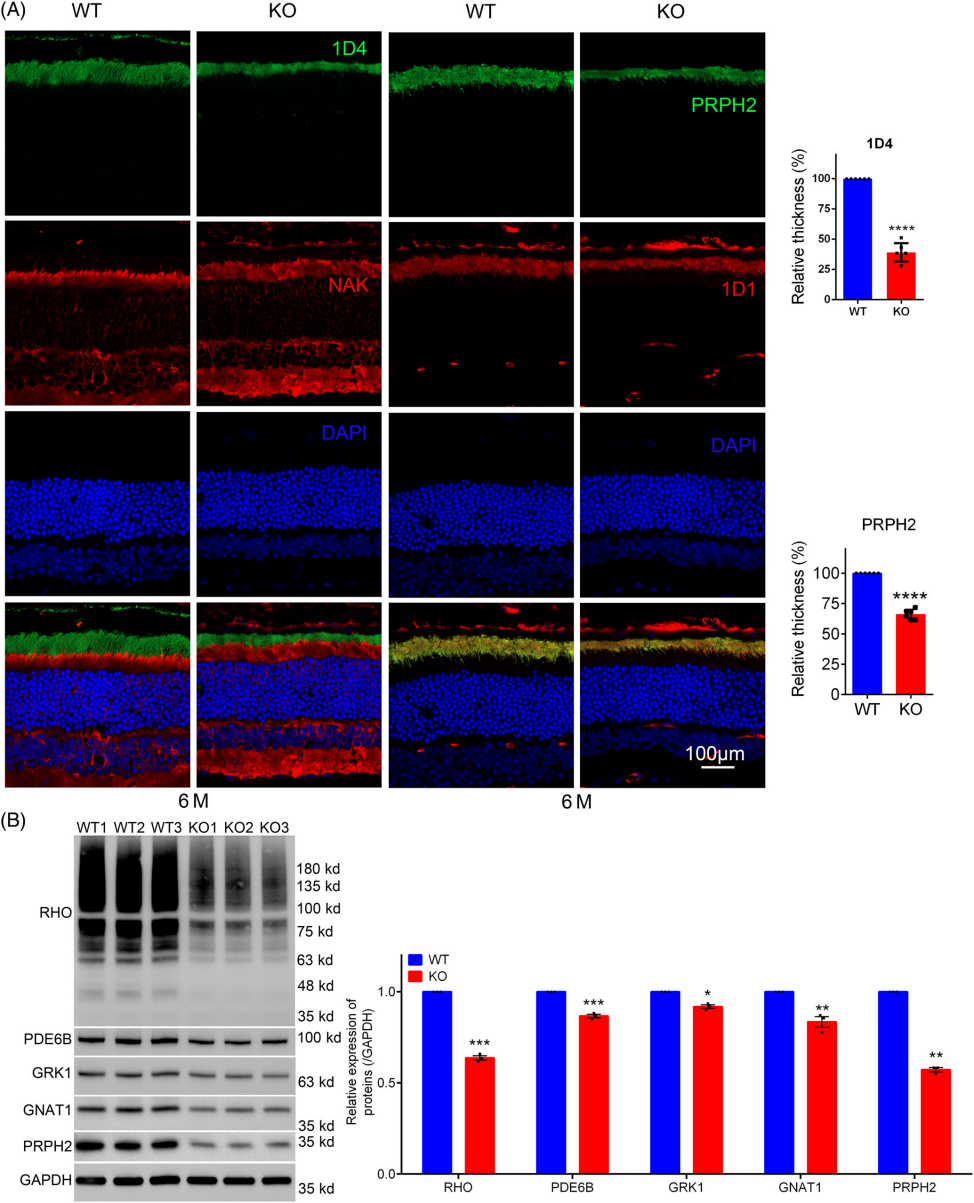
Immunofluorescence staining (rod) and western blotting revealed *Tmem184b* knockout (KO) mice suffered from severe photoreceptor degradation. (A) anti‐rhodopsin (1D4) and peripherin 2 (PRPH2) antibodies were used to stain outer segments of rods, anti‐NaK ATPase and CNGA1 (1D1) were used to stain cell bodies of rod photoreceptors, respectively. Rod‐related statistical charts were arranged in the right panel. (B) Immunoblotting and quantitative comparison of phototransduction‐related proteins. Protein expression level were normalized according to GAPDH (*n* = 3). White scale bar showed the length of corresponding size above it. Data were presented as mean ± SEM. ****p < 0.0005, ***p < 0.001, **p < 0.01, *p < 0.05.

Meanwhile, 1D4 and PRPH2 staining showed that outer segments of KO mice became much thinner than that of WT (Figure 5A, left). Statistical analysis of thickness of rods outer segments were showed in the right panel of Figure 5A. Severe decline in scotopic ERG and significant ONL cells loss promoted us to explore molecular changes within photoreceptor cells. Photo‐transduction is the initial step of visual perception in photoreceptor cells of retina. Various proteins, such as rhodopsin (RHO), phosphodiesterase 6B (PDE6B), G protein‐coupled receptor kinase 1 (GRK1), G protein subunit alpha transducin 1 (GNAT1) and PRPH2, played essential roles in this process.^23^, ^24^, ^25^, ^26^, ^27^ Fresh retinal proteins were extracted and subjected to western blot (WB) analysis. Results showed significant reduction in RHO, PDE6B, GRK1, GNAT1 and PRPH2 protein levels in KO retinas (Figure 5B), suggesting that deletion of *Tmem184b* led to diminished levels of these phototransduction‐related proteins.

### 2.5 Retinal inflammation and cell death in inner retina demonstrated extensive degeneration of multiple cell types

**FIGURE 6:**
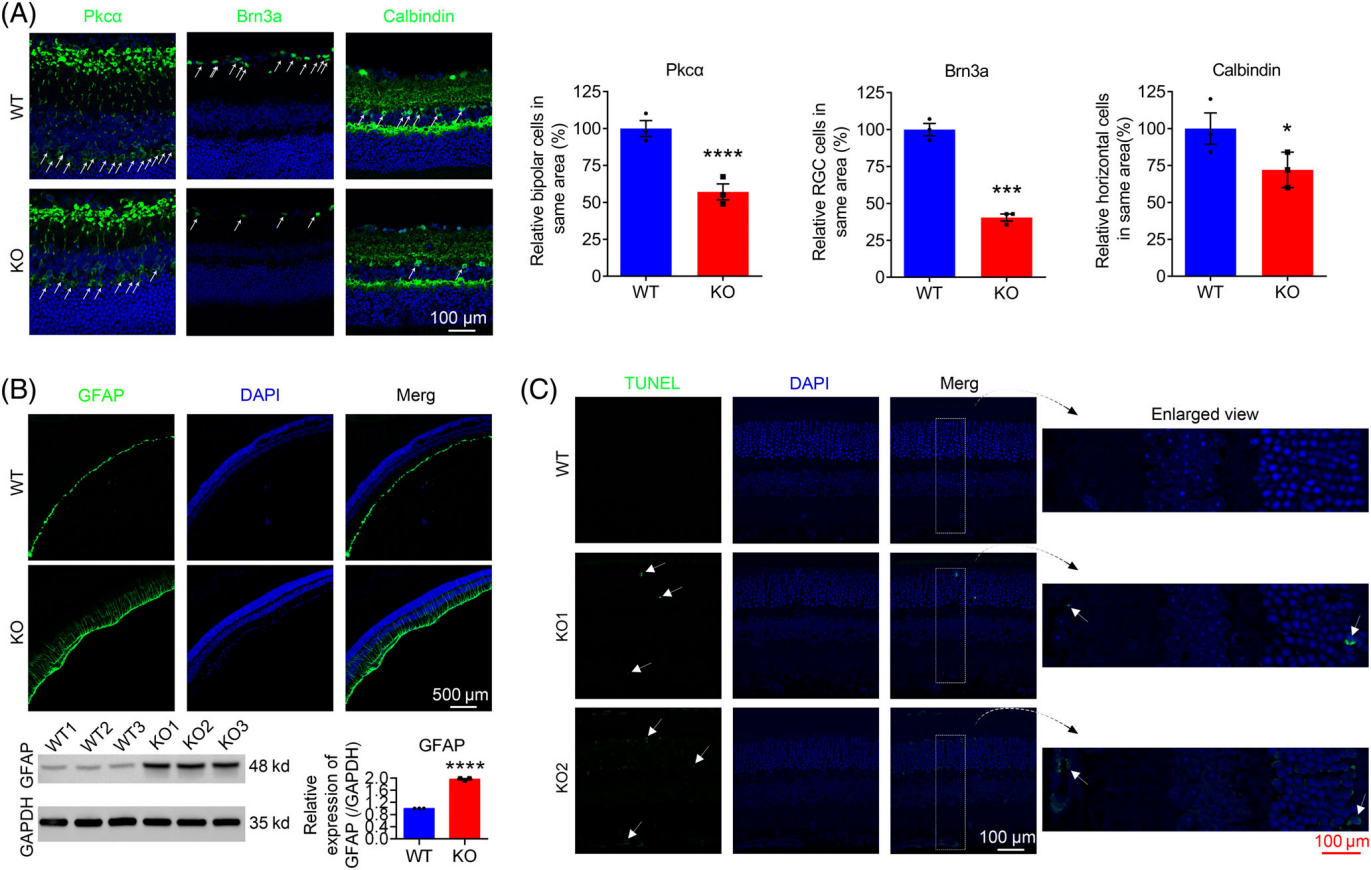
Extensive inflammation and cell death were observed in *Tmem184b* knockout (KO) mice. (A) Immunofluorescence (IF) staining on frozen sections (left) and cell counts (right) showed bipolar, RGC and horizontal cells degeneration. (B) GFAP IF staining (left) and western blotting (right) showed GFAP upregulated indicating inflammation was activated. Relative expression of GFAP from western blotting was normalized according to GAPDH and showed under its blotting bands. (*n* = 3) (C) TUNEL (terminal deoxynucleotidyl transferase‐mediated biotinylated UTP nick end labelling) staining on frozen sections detected positive cell death in retina. Enlarged images were showed on the right side of the original view. White arrows showed positive cells and scale bars showed the corresponding length in these photos. Data were presented as mean ± SEM. ****p < 0.0005, ***p < 0.001, *p < 0.05.

Anti‐Pkcα (protein kinase C α), brain specific homeobox 3a (Brn3a) and calbindin antibodies were used to visualize bipolar cells, retinal ganglion cells and horizontal cells, respectively. Immunostaining analysis of retinal frozen sections showed significant reduction of cell numbers was observed in bipolar, RGC and horizontal cells (Figure 6A). Furthermore, as a symbol of retinal dystrophy, retina inflammation, which was characterized with the activation of GFAP‐positive glia cells,^28^, ^29^ was prominent, as revealed by retina frozen section IF staining and WB using GFAP antibody in KO mice (Figure 6B), suggesting that inflammation played a role in *Tmem184b* KO‐induced retina degenerating process.

To detect if cell death played a role in *Tmem184b* KO model, TUNEL (terminal deoxynucleotidyl transferase‐mediated biotinylated UTP nick end labelling) assay was performed on retina frozen sections of 6 months old tested mice. As showed in Figure 6C, TUNEL‐positive cells were detected in several layers of the KO retinas, including inner nuclear layer (INL) and ONL.

To visualize more detailed astrocytosis in the retina, GFAP and IB4 antibodies were used to label retinal whole mounts form *Tmem184b* KO. Additionally, Ionized calcium‐binding adapter molecule 1 (IBA1) and macrosialin (CD68) antibodies were used as microglia and pro‐inflammatory markers to stain the retina whole mounts. The results were exhibited in Figures S3 and S4, respectively. Compared with WT controls, more GFAP‐positive cells were present in KO group, and their active cell morphology indicated astrocytosis (Figure S3). And more active microglia cells judging from multiple pseudopodia cells' morphology were found in retinal whole mounts of KO mice, while inactivated microglia cells were present in WT mice. These results indicate overall inflation response upon *Tmem184b* deletion.

### 2.6 Transcriptome sequencing revealed *Tmem184b* KO‐induced genes related to several bioprocesses down‐regulation

**FIGURE 7:**
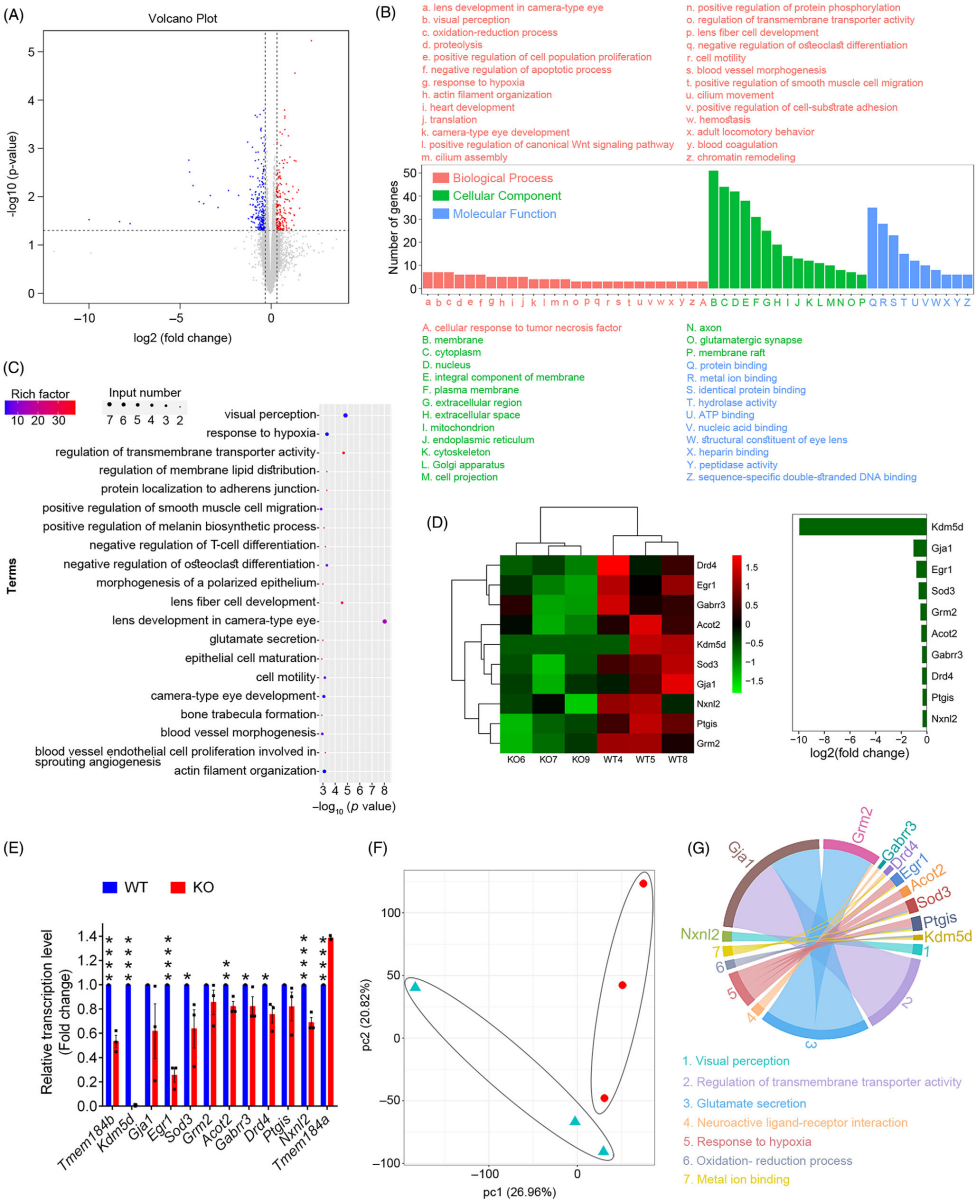
Transcriptome sequencing revealed downstream genes affected by *Tmem184b* deficiency. (A) Volcano plot displaying genes were differentially transcribed between wild‐type (WT) and knockout (KO) groups and their statistical significance (fold changes ≥1.5 and *p* < 0.05). Up‐ and down‐regulated genes were plotted in red and blue, respectively. (B) Gene Ontology (GO) enrichment analysis. Down‐regulated genes were classified into several categories related to different biological processes (red), cellular components (green) and molecular functions (blue) and were plotted to *y*‐axis by the numbers of these genes in the same one category. (C) GO‐enriched pathways, enriched factors were showed in gradient colour from blue (less) to red (more), and gene input numbers of each pathway were plotted as different sizes of the circular spots in the up left corner. (D) Heat maps of GO enriched 10 most down regulated genes. (E) qPCR verification columns confirmed these 10 most down regulated genes and *Tmem184a* (a paralog of *Tmem184b*) was up‐regulated. (F) PCA results visualized the correlation between KO and WT groups' gene‐expressing patterns. (G) Chord diagram exhibited the relationship of these 10 genes and their related biological processes. ****p < 0.0005, **p < 0.01, *p < 0.05 (*n* = 3). PCA, principal component analysis.

To investigate the internal molecular mechanisms of retinal degeneration induced by *Tmem184b* deficiency, RNA‐sequencing analysis was performed on three KO versus three WT mice. As showed in Figure 6, 191 and 229 genes in total were found to be up or down‐regulated in KO compared with their littermate controls, respectively. Volcano plot (Figure 7A) indicated that those genes with fold change greater than two concentrated on the down‐regulation group. Gene Ontology (GO) enrichment analysis showed genes related to visual perception, response to hypoxia were enriched among top three biological processes (Figure 7B–D). Enriched top three involved in cell components were related to genes expressing in membrane, cytoplasm and nucleus, while top three involved in molecular functions were related to protein binding, metal ion binding and identical protein binding (Figure 7B). Pathways related to visual perception, response to hypoxia and regulation of transmembrane transporter activity were the top three affected pathways (Figure 7C). Heat maps of the RNA‐sequencing (RNA‐seq) results showed that multiple core genes in the visual perception, response to hypoxia pathways and regulation of transmembrane transporter activity were significantly down‐regulated (Figure 7D). Furthermore, quantitive polymerase chain reaction (qPCR) analysis confirmed the abovementioned RNA‐seq pathway results, ultimately indicating that *Tmem184b* may affect retinal homeostasis through these pathways in the retina (Figure 7E). Additionally, qPCR also revealed that *Tmem184a*, an important paralog of *Tmem184b*, was up regulated which may be related to the reverse feedback regulation (Figure 7E). Principal component analysis was used to visualize the correlation between gene expression in KO and WT groups (Figure 7F). Gene expression patterns of two groups were quite different. In order to exhibit the down‐regulated genes and their related bioprocesses, a chord diagram was created and displayed in Figure 7G. These 10 most down‐regulated genes belong to one or more biological processes such as visual perception, response to hypoxia, regulation of transmembrane transporter activity, glutamate secretion, oxidation–reduction process, neuroactive ligand‐receptor interaction and metal ion binding.

## 3 DISCUSSION

In this study, we investigated the in vivo roles of *Tmem184b* in the retina using *Tmem184b* KO mouse model. *Tmem184b* KO mice exhibited diminished a‐ and b‐ wave amplitudes in dark adaption and b wave amplitudes in light response, indicating that impaired photoreceptors function. Meanwhile, fundus imaging and H&E staining showed widespread damage in *Tmem184b* KO mice retina. Additionally, IF staining of different retinal componential cells also indicates extensive lesions including photoreceptors, bipolar cells, horizontal cells and RGCs damages. TUNEL and GFAP staining indicated cell death and inflammation in the retina of KO mice, highlighting its crucial role in retinal function and survival.

RNA‐sequencing was used to explore the preliminary pathogenesis mechanism, which manifested that the most significantly regulated gene is *Kdm5d*, which encodes an H3K4 histone demethylase. And *Kdm5d* gene was reported to be involved in cancer,^30^, ^31^, ^32^, ^33^ cardiomyopathy,^34^, ^35^ renal cell carcinoma,^36^ neurodevelopmental diseases^37^ and Huntington's disease.^38^ As a Y‐encoded demethylase, *Kdm5d* is involved in sexually dimorphic gene expression,^39^ plays a role in sex determination^40^ and displays sex differences in many diseases.^30^, ^41^ And this sex difference also has been exhibited in *Kdm5d* related nervous injury responses.^42^ It is interesting that *Kdm5d* showed high expression level in the retina (https://www.proteinatlas.org/ENSG00000012817-KDM5D/single+cell+type/eye). *Kdm5d* should be given more attention to further study on RD.

Additionally, the down‐regulated genes in the response to hypoxia pathway may indicate a possible mechanism of action to *Tmem184b* KO‐induced retina degeneration. Biological processes of response to hypoxia played crucial roles in maintaining normal eye physiology.^43^, ^44^ In this pathway, *Egr1*, *Acot2*, *Sod3* and *Ptgis* genes were down‐regulated. *Egr1*, a transcriptional regulator, belongs to the EGR family of C2H2‐type zinc‐finger proteins. Its target genes are required for differentiation and mitogenesis.^45^ And paired like homeodomain (RIEG/PITX) homeobox family member, *Pitx2* was previously reported to be related with glaucoma.^46^ Its same family member gene, *Pitx1*, is a down streaming‐regulated gene of *Egr1*.^47^ *Acot2* gene encodes an acyl‐CoA (cozenzyme A) hydrolase plays important role in lipids metabolism^48^ while *Sod3* gene encodes a superoxide dismutase which catalyses superoxide radicals into hydrogen peroxide and oxygen, protecting eye, neuro and other tissues from oxidative stress.^49^ And *Ptgis* gene's full name is *Prostaglandin I2 synthase* gene, it encodes an enzyme member of the cytochrome P450 superfamily, which catalyses the conversion of prostaglandin H2 to prostacyclin, playing very important role in synthesis of cholesterol, steroids and other lipids.^50^ These findings indicate that response to hypoxia pathway plays a role in *Tmem184b* KO‐induced retina degenerating process.

Retinal angiogenesis defects may be induced by various inherited factors,^51^, ^52^ leading to retinal hypoxia. However, no significant retinal vascular structural anomaly was found in *Tmem184b* KO mice. As a transmembrane protein, TMEM184b was reported to be a suspected transporter of taurine,^21^ a known strong antioxidant, which played very important role in the nervous system, especially in the retina.^53^ Taurine transport and metabolism should be paid enough attention to in the further studies. Overall, our data demonstrate a novel regulating mechanism of TMEM184B in retinal degeneration, providing a novel potential target for RD therapy development.

## 4 MATERIALS AND METHODS

### 4.1 Animal models

All the animal study protocols were reviewed and approved by the Animal Care and Use Committee of Sichuan Provincial People's Hospital and followed the guidance of the Association for Research in Vision and Ophtaalmology (ARVO) statement for the use of animals in ophthalmic and vision research. Animals were housed in a room with a constant temperature of 25°C, 12 h light/dark cycle and unrestricted feed and drinking water. Over dosage intraperitoneal injection of ketamine anaesthetics and followed by cervical dislocation was used for mouse euthanasia.

CRISPR/Cas9 method was used to knock out *Tmem184b* gene in C57BL/6N background (*Tmem184b* ^*em1zxj*^, named *Tmem184b* KO for convenience). The knock out strategy was showed in Figure 1, and the following gRNA target sequences were selected: GCTCCCGTGCGCTCAGTGAGTGG and AGAGCACAGGGTCGAGTGTAAGG. A 4002 bp DNA fragment flanking exons 4, 5 and 6 was deleted in the *Tmem184b* KO allele. The KO mice were bred to C57BL/6J for five generations to remove *rd8* mutation. Because HET animal phenotype was similar to WT ones (Figure S2A), and female animals were mainly used for expanding the breeding population, for convenience, only male KO and WT animals were selected for experiments followed by.

### 4.2 Genotyping

Mouse genomic DNA samples were extracted from tail tips and genotyped by PCR. A pair of primers was designed as primer F1: AAGGGAAGGGTGTAAGCTCTGG and primer R1: CAGGCTGACGGAGATGTTGTAG to identify the genotypes of new born mice. PCR master mix (Invitrogen, CA, USA) was used to amplify the *Tmem184b* gene sequence according to the manufacturer's instructions. PCR reactions were initialled by 5 min 95°C step and followed by 32 cycles of 95°C for 15 s, 60°C for 30 s and 72°C for 30 s. PCR products were separated by 3% agarose gel electrophoresis in order to identify homozygous KO, heterozygous KO or WT alleles by different bands (Figure 1).

### 4.3 ERG and fundus imaging

ERG was performed following the protocol of our former report.^54^ Dark‐adapted (scotopic) ERG was performed firstly. In a darkroom, animal was anaesthetised with an intraperitoneal injection of mixed xylazine (80 mg/kg) and ketamine (16 mg/kg) saline solution after overnight dark adaption. Then testing animal was connected to an instrument set of Roland electroretinogram recorder and Ganzfeld Q450 stimulator (Roland Consult, Heidelberger, Germany) with needle type subcutaneous reference electrodes and circular recording electrodes on corneal. Scotopic a‐ and b‐ wave was recorded under 3.0 and 10.0 cds/m^2^ light stimulation. Then, a 10 min white colour light adaptation (25 cds/m^2^) were performed before recording the photopic 3.0 ERG (stimulated by 3.0 cds/m^2^ white flashes). A‐ and b‐ wave amplitudes were recorded and counted for average.

Under anaesthetised condition, fundus photographs were obtained by using a Phoenix MICRON IV system (Phoenix Research Labs, Bend, OR, USA) according to the manufacturer's instruction.

### 4.4 Optical coherence tomography

In order to assess the detailed retina structure in vivo, OCT was performed by Phoenix MICRON IV system (Phoenix Research Labs, Bend, OR, USA), according to the manufacturer's instruction.

### 4.5 Rotarod test

Rotarod test was performed as described before^55^ to investigate if there is any motor deficits. The test animals were placed on the rolling rod (4 rpm) of the rotarod test device (Unibiolab, Shanghai, China). The latency to fall off was recorded and subjected to statistical analysis.

### 4.6 Histological analysis

Enucleated eyes of euthanized mice were fixed overnight in 4% paraformaldehyde phosphate buffer, embedded in paraffin and then cut into 5 μm sections. H&E staining were performed as described previously on sections that encompassed the optic nerve (ON).^56^

### 4.7 Frozen sections and IF staining

Eyeballs of euthanized mice were collected and lens were removed before fixed for 1 h at 4°C in 4% paraformaldehyde phosphate buffer. Optimal cutting temperature solution were used to embed the eyes in same orientation to ensure the same eccentricity of sections. Thereafter, 10 μm thick sections were cut encompassing the ON. One hour blocking and permeabilization step was performed in 10% normal donkey serum and 0.2% Triton X‐100 in phosphate buffer. And then frozen sections were labelled with one of the primary antibodies overnight at 4°C, which were showed in Table S1. After rinsing in PBS for three times, goat anti‐mouse/rabbit secondary antibody labelled with Alexa Fluor 594/488 (1:500 dilution, Invitrogen, Waltham, MA, USA) and 4',6‐diamidino‐2‐phenylindole (DAPI) (Sigma, St Louis, MO, USA) were added for counterstaining the different types of cells and their nuclei at room temperature. One‐hour later, sections were rinsed again for three times before mounting. Zeiss LSM 900 confocal scanning microscope and ZEN 2.3 imaging software (Zeiss, Oberkochen, Germany) was used for imaging and quantifying the fluorescence intensity, respectively.

### 4.8 Survived cone quantification in retinal whole mounts

Retinas were dissected, flattened and fixed in 4% PFA for 24 h at 4°C. After washing for three times with PBS, a solution with 1% bovine serum albumin, 0.5% Triton X‐100 and polyclonal rabbit anti‐L/M‐Opsin antibody in PBS was incubated for 12 h at 4°C. The retinas were then incubated with Alexa Fluor 594‐conjugated peanut agglutinin (PNA) and an AlexaFluor 488‐conjugated goat anti‐rabbit secondary antibody (Invitrogen, Waltham, MA, USA, 1:250 dilution) for 4 h at room temperature. Three times of washes were applied before mounting. And several areas from the optic disc of these retinas were selected and photographed for cone quantification. Cones were counted manually in each selected sector to determine the average cone density (cones/mm^2^).

In order to illustrate astrocytosis in context with the overall retinal structure, GFAP and Alexa Fluor™ 594 conjugated lectin B4 (IB4) were used together to stain the astrocytes and retinal blood vessel in retinal whole mounts, respectively. IBA1 and CD68 were used as microglia and pro‐inflammatory markers to stain the retinal whole mounts.

### 4.9 TUNEL assay

The TUNEL assay was used to detect the apoptotic cell death according to the manufacturer's instruction (Roche Diagnostics, Indianapolis, IN, USA) on frozen sections. Images were captured by Zeiss LSM 800 confocal scanning microscope.

### 4.10 Western blotting

Tissues lysis immunoprecipitation assay buffer contains a protease inhibitor cocktail (Roche, Redwood City, CA, USA) and a phosphatase inhibitor (Roche, Redwood City, CA, USA). The extracted total protein concentration was determined by a DC Protein Assay according to the manufacturer's instructions (Bio‐Rad, Hercules, CA, USA). Protein was diluted to equal amounts and separated by electrophoresis on sodium dodecyl sulfonate polyacrylamide gels and transferred to NC membranes (Millipore, Billerica, MA, USA). After blocking in 8% non‐fat dry milk in Tris‐buffered saline (TBS) solution with Tween® 20 detergent for 2 h at room temperature, the membranes were incubated with the primary antibodies in blocking solution overnight at 4°C. The primary antibodies used for WB are shown in Table S2. Anti‐mouse or anti‐rabbit horseradish peroxidase (HRP)‐conjugated secondary antibodies (1:5000; Bio‐Rad, Hercules, CA, USA) along with SuperSignal™ West Pico PLUS Chemiluminescent Substrate (Thermo, Waltham, USA) were used for visualization. The relative intensity of the immune reactive bands was quantified by ImageJ software. The proteins intensity was normalized using glayceraldehyde‐3‐phosphate dehydrogenase (GAPDH) as an internal reference. At least three independent repeats were conducted. And one typical blot was presented.

### 4.11 RNA‐sequencing

RNA‐sequencing was performed on three pairs of WT or KO retinas at 4 months of age. After harvest, retinas were collected, frozen immediately and sent to Seqhealth Corporation Inc. (Wuhan, China) for high‐throughput RNA‐seq on an Illumina HiSeq 2500 platform generating 150 bp paired‐end reads. All gene expression values from RNA‐seq were converted to related log2 values for further analysed. A *p* value no more than 0.05 was considered to be significant.

### 4.12 RNA extraction and Reverse transcription‐quantitive polymerase chain reaction (RT‐qPCR)

Retinal total RNA was extracted by using TRIzol reagent (Sigma, Saint Louis, MO, USA) according to manufacturer's instruction. First‐strand cDNA was synthesized by using an iScript cDNA Synthesis Kit (Bio‐Rad, Hercules, CA, USA). Quantitative PCR was performed by using iTaq SYBRMix (Bio‐Rad, Hercules, CA, USA) and on a CFX384 Touch Real‐Time PCR Detection System (Bio‐Rad, Hercules, CA, USA). Primers were designed by using Primer3Plus online tools. Primer sequences were listed in Table S3. All target genes were normalized to *Gapdh* mRNA levels, and the fold change was calculated by delta–delta Ct analysis.

### 4.13 Statistical analysis

Statistical analysis was performed by GraphPad Prism 6 software (GraphPad Software, Boston, MA, USA). A normal distribution test was firstly used to determine if the data was normally distributed by Shapiro–Wilk test. If yes, statistical significance was determined by Student's *t*‐test or ANOVA. Otherwise, a non‐parametric statistic was used. *p* < 0.05 was considered as statistical significant.

## AUTHOR CONTRIBUTIONS

Conceptualization and methodology were provided by Xianjun Zhu and Yeming Yang; investigation and formal analysis were finished by Guo Liu, Junkai Tan, Tiannan Liu, Xiaoyan Jiang, Kuanxiang Sun and Wenjing Liu; original draft was written by Guo Liu, Yeming Yang, Xuyang Liu and Xianjun Zhu; revision were done by all authors; daily supervision, project administration and funding acquisition were proved by Xianjun Zhu.

## FUNDING INFORMATION

This study was supported by the National Natural Science Foundation of China (82371083, 82070963, 82121003), the Department of Science and Technology of Sichuan Province (2023ZYD0172), the Chinese Academy of Medical Sciences (CAMS) Innovation Fund for Medical Sciences (CIFMS) (2019‐12M‐5‐032), the Huanhua Scholar Program and the Sichuan Provincial People's Hospital Postdoctoral Fund (2022BH02).

## CONFLICT OF INTEREST STATEMENT

These authors declare no conflicts of interest.

## IRB STATEMENT

All the animal study protocols were reviewed and approved by the Animal Care and Use Committee of Sichuan Provincial People's Hospital and followed the guidance of the ARVO statement for the use of animals in ophthalmic and vision research.

## Supporting information


**Data S1:** Supporting Information.

## Data Availability

The data that support the findings of this study are available from the corresponding author upon reasonable request.
