# Supplementary material for: Deletion of Transmembrane protein 184b leads to retina degeneration in mice
Source: Cell Prolif. 2024 Oct 7;58(2):e13751. doi: 10.1111/cpr.13751 (PMC11839198; doi:10.1111/cpr.13751)
Supplement: Supplementary file 1 — Data S1: Supporting Information. [file CPR-58-e13751-s001.pdf]

Deletion of *Tmem184b* leads to retina degeneration in mice

Guo Liu[1,2#], Tiannan Liu[1#], Junkai Tan[3#], Xiaoyan Jiang[1], Yudi Fan[1], Kuanxiang Sun[1], Wenjing Liu[1], Xuyang Liu[3*], Yeming Yang[1*], Xianjun Zhu[1-5*]

Supplementary data include figures S1-S4 and table S1-S3.

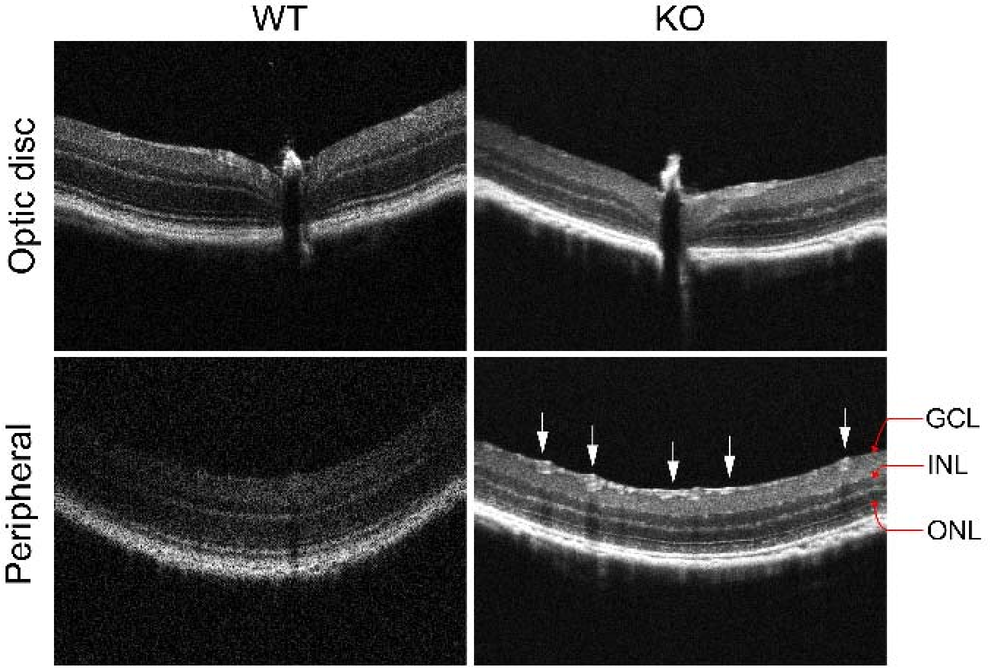

Figure S1. Optical coherence tomography of 6 months old *Tmem184b* KO mice and control.

White arrows showed high reflection points in GCLs of KO mice.

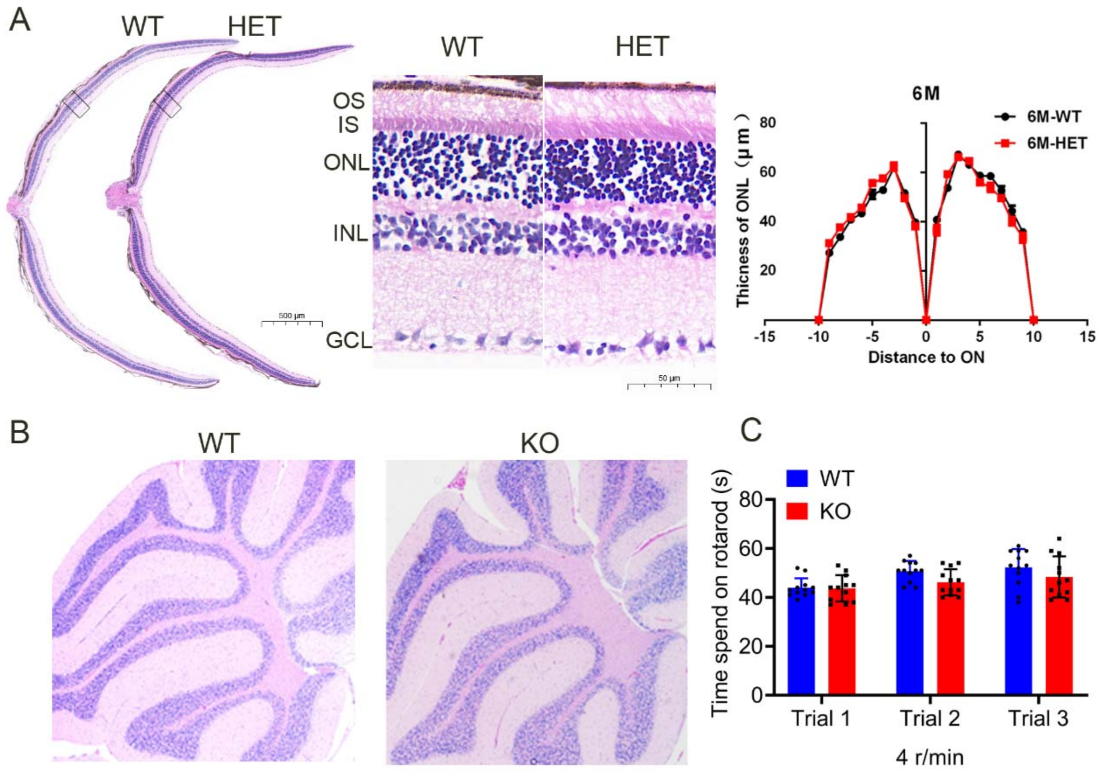

Figure S2. Rotarod test and H&E staining in retinal and cerebellar paraffin sections of Six months old *Tmem184b* KO and control mice.

A: Retinal H&E results revealed there was no significant difference between six months old WT and HET mice. B: No significant structural changes in cerebellum were found in Six months old *Tmem184b* KO mice. C: No motor deficits were detected in six months old *Tmem184b* KO mice.

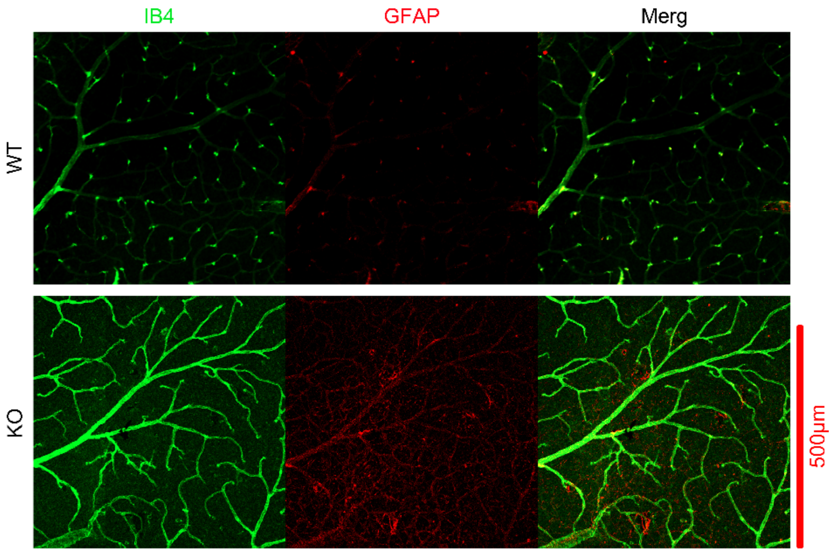

Figure S3. GFAP and IB4 staining with retinal whole mounts.

GFAP positive cells (red) arrangement and their active cell morphology were detected more in the KO group. IB4 antibody marked the retinal blood vessels in green. The red bar showed the size of 500μm.

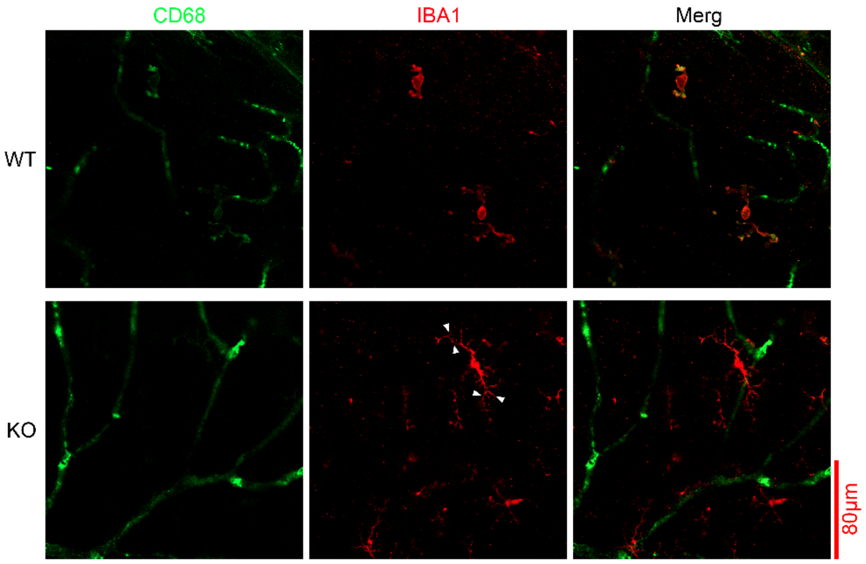

Figure S4. Microglia cells stained by IBA1 antibody.

Active microglia cells judging from multiple pseudopodia cells' morphology were more frequently found in retina whole mounts of KO mice, while inactivated microglia cells were more frequently found in WT mice. The red bar showed the size of 80μm.

Table S1 Antibodies used in immunofluorescence staining.

Antibody	Dilution rate	Species	Company	Cat. No.
Anti-GFAP	1:200	Mouse	Abcam, MA, USA	ab169276
Anti-Rhodopsin (1D4)	1:200	Rabbit	Proteintech, IL, USA	11308-1-AP
Anti-PRPH2	1:200	Rabbit	Abcam, MA, USA	ab84036
Anti-NaK ATPsae	1:500	Mouse	Abcam, MA, USA	ab18230
Anti-PKCα	1:200	Mouse	Thermo MA USA	MA1-157
Anti-Calbindin	1:200	Rabbit	CST, MA, USA	D1I4Q
Anti-Brn3a	1:200	Rabbit	Abcam, MA, USA	ab245230
Anti-Cone Arrestin	1:200	Rabbit	Sigma MO USA	ab15282
Anti-L/M Opsin	1:500	Rabbit	Sigma MO USA	ab5405
Anti-CNGA1 (PMc1D1)	1:200	Mouse	Abcam, MA, USA	ab253296
Alexa Fluor™ 594 conjugated PNA	1:200	_	Thermo, MA, USA	L32459
Alexa Fluor™ 594 conjugated lectin B4 (IB4)	1:200	_	Thermo, MA, USA	I21413
Anti-IBA1	1:200	Rabbit	Thermo, MA, USA	PA5-88519
Anti-CD68	1:200	Mouse	Thermo, MA, USA	14-0688-82

Table S2 Antibodies used in Western blotting.

Antibody	Dilution rate	Species	Company	Cat. No.
Anti-Rhodopsin	1:2000	Mouse	CST, MA, USA	3700
Anti-GNAT1	1:1000	Rabbit	Abcam, MA, USA	ab21679
Anti-PDE6B	1:2000	Rabbit	Thermo, MA, USA	T13343
Anti-PRPH2	1:2000	Rabbit	Abcam, MA, USA	ab84036
Anti-GRK1	1:1000	Rabbit	Abcam, MA, USA	ab54460
Anti-GAPDH	1:2000	Rabbit	Abcam, MA, USA	ab14181
Anti-GFAP	1:2000	Mouse	Abcam, MA, USA	ab169276

Table S3 Primers used in quantitative PCR validation.

Gene name	Primer info
Tmem184b	Forward primer: AACCACAACAGCTTCACCTAGC
	Reverse primer: TGGCAAGTGATGAGCAGAGC
Kdm5d	Forward primer: CCAGGATCTGACGACTTTCTACC
	Reverse primer: TTCTCCGCAATGGGTCTGATT
Gja1	Forward primer: ACAGCGGTTGAGTCAGCTTG
	Reverse primer: GAGAGATGGGGAAGGACTTGT
Egr1	Forward primer: ACTTTGCGCCTACAATTCAGG
	Reverse primer: AACTTGCCAGGGAATGGAACT
Sod3	Forward primer: CCTTCTTGTTCTACGGCTTGC
	Reverse primer: TCGCCTATCTTCTCAACCAGG
Grm2	Forward primer: GCTCCCACAGCTATCACCG
	Reverse primer: TCATAACGGGACTTGTCGCTC
Acot2	Forward primer: GTTGTGCCAACAGGATTGGAA
	Reverse primer: GCTCAGCGTCGCATTTGTC
Gabrr3	Forward primer: AGGAGACAGTTCAGAAGTGGG
	Reverse primer: GCCAGCACACGTACTTATCAA
Drd4	Forward primer: GCCTGGAGAACCGAGACTATG
	Reverse primer: CGGCTGTGAAGTTTGGTGTG
Pigis	Forward primer: ACAGCATCAAACAATTTGTCGTC
	Reverse primer: GCATCAGACCGAAGCCATATCT

Nxnl2	Forward primer: GTGGTAGCTTTGTACTTTGCGG
	Reverse primer: CCGTCTGCCGACACGAAAA
Gapdh	Forward primer: TTTGCACTGGTACGTGTTGAT
	Reverse primer: TGGATTTGGACGCATTGGTC
Tmem184a	Forward primer: ATGAGGAATGCGTCAGGGTTT
	Reverse primer: AGGAACGTAGGTGGGAGTAGA
